# Endometrial hypoperfusion: the missing link in refractory thin endometrium

**DOI:** 10.3389/frph.2025.1732672

**Published:** 2025-12-03

**Authors:** I. Weizel, D. Lasri, A. Hersko Klement, Y. Bentov

**Affiliations:** 1 Faculty of Medicine, Hebrew University of Jerusalem, Jerusalem, Israel; 2 Division of Obstetrics and Gynecology, Hadassah-Hebrew University Medical Center, Jerusalem, Israel; 3 Department of Obstetrics and Gynecology, Hadassah Mount Scopus-Hebrew University Medical Center, Jerusalem, Israel

**Keywords:** thin endometrium, refractory endometrium, endometrial blood supply, vasculature, doppler, VEGF, hypoxia, management

## Abstract

Persistently thin endometrium remains one of the hardest challenges in reproductive medicine, often linked to implantation failure and poor pregnancy outcome. Growing evidence implicates deficient uterine blood supply in the pathogenesis of an inadequately thin endometrial lining. This narrative review synthesizes current knowledge on how endometrial vascularization influences endometrial thickness (EMT) and receptivity. We outline the normal endometrial structure and vascular physiology, then examine the pathophysiological features of thin endometrium, highlighting mechanistic studies that demonstrate impaired angiogenesis, reduced microvessel density, and tissue hypoxia in thin endometrial tissue. Doppler ultrasound studies consistently show that women with thin endometrium have reduced endometrial and subendometrial blood flow and higher resistance indices. Clinical correlations indicate that poor endometrial perfusion is associated with lower implantation and pregnancy rates in assisted reproduction. We review diagnostic tools for assessing endometrial perfusion, including two-dimensional (2D) and three-dimensional (3D) Doppler ultrasound measures of uterine and subendometrial blood flow. Therapeutic strategies aiming to improve uterine blood supply, such as vasoactive medications, intrauterine infusion of platelet-rich plasma (PRP), stem cell therapies, and other angiogenic treatments, often result in a marginal improvement of EMT and pregnancy outcomes. While these interventions show promise, limitations include small sample sizes and heterogeneous study designs. We discuss future directions, emphasizing the need for larger trials and a deeper understanding of angiogenic signaling in the endometrium. In conclusion, converging evidence supports poor endometrial blood supply as a key contributor to persistently thin endometrium. Future therapies that specifically target enhancement of endometrial blood supply may prove to be effective tools for improving endometrial growth and fertility outcomes.

## Introduction

Numerous patients worldwide struggle with thin EMT that often greatly delays and sometimes prevents the achievement of a pregnancy. Persistently thin endometrial lining has been recognized as a poor prognostic factor for fertility treatment outcome, independent of embryo quality (1, 2). EMT of <8 mm was identified to be associated with a lower rate of implantation in ART and non-ART fertility treatments (3–5). Furthermore, if implantation occurs despite the presence of a thin endometrial lining the resulting pregnancy is prone to complications (6). In a large retrospective study (*N* = 244,000 cycles) the incidence of thin endometrium (<8 mm) among patients undergoing embryo transfer (ET) was 15% (5). However, since most ET cycles that fail to achieve adequate EMT are cancelled due to their well-known poor outcomes, the true incidence is likely underestimated. In non-ART treatment cycles, by contrast, estimated incidence ranges from 38% to 66% (7). The management of thin endometrium includes hysteroscopic evaluation to rule out endometrial adhesions and endometritis. Once ruled out, treatment commonly involves intensifying estrogen stimulation. However, if EMT remains inadequate, clinicians often resort to an array of empirical solutions including change of protocol as well as use of adjuvants including Aspirin, Sildenafil, G-CSF, PRP, Growth Hormone, Pentoxifylline-Tocopherol and others (8). None of these interventions, when compared in an RCT resulted in a persistently improved EMT (8).

### Endometrial histology and angiogenesis

**Figure 1:**
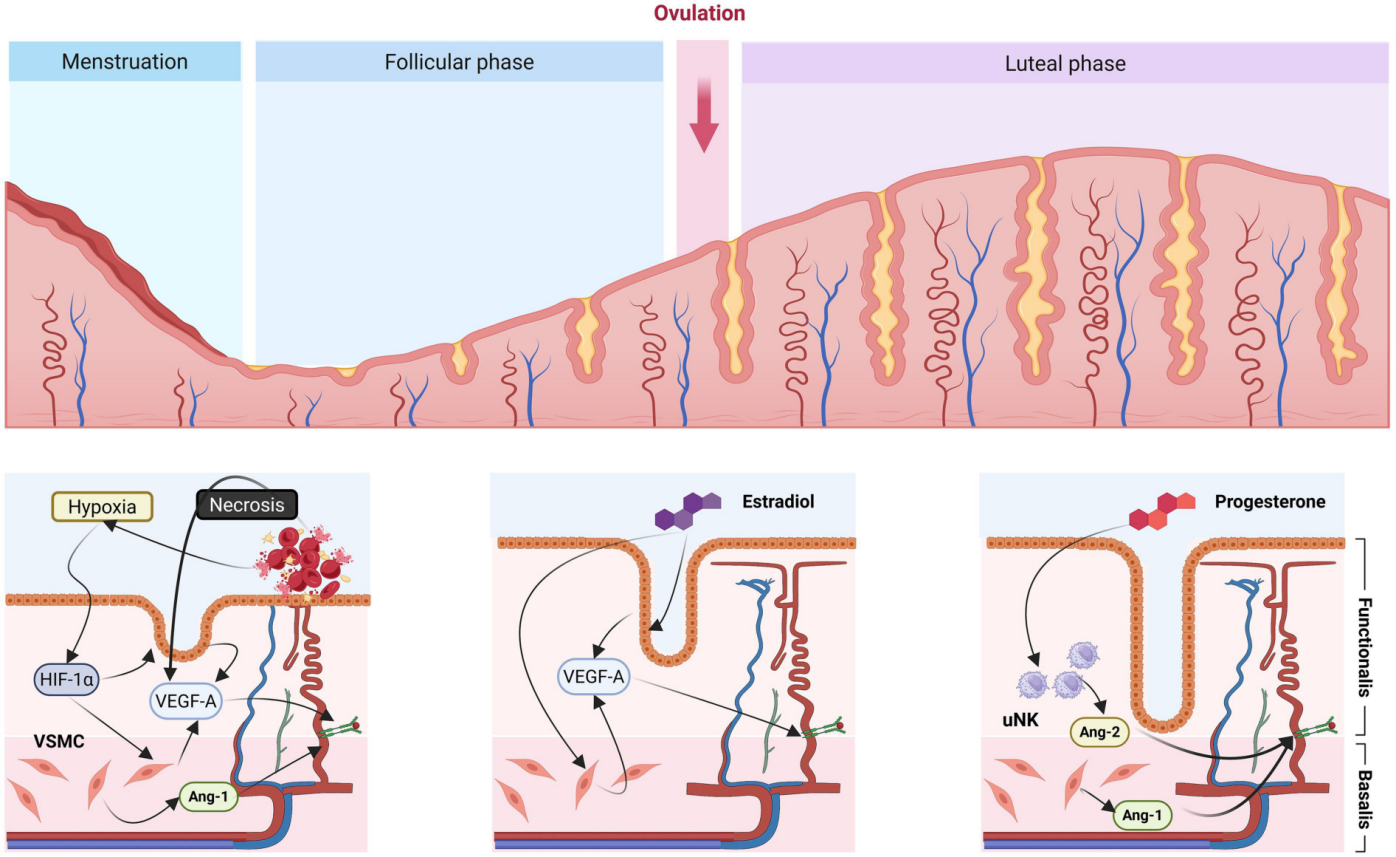
Regulation of endometrial angiogenesis throughout the menstrual cycle. Schematic representation of the cyclical remodeling of the endometrial vasculature and its hormonal regulation. The upper panel illustrates the morphological evolution of the endometrium across the menstrual, follicular, and luteal phases. During **menstruation**, tissue shedding and transient **hypoxia** activate angiogenic signaling through **HIF-1α** and **VEGF-A**, initiating endothelial repair and vascular smooth muscle cell (VSMC) activation. In the **follicular phase**, rising **estradiol** stimulates endothelial proliferation and vascular expansion via upregulation of **VEGF-A** and other pro-angiogenic mediators. In the **luteal phase**, **progesterone** promotes vascular stabilization and maturation, supported by **uterine natural killer (uNK) cells** secreting **angiopoietin-2 (Ang-2)**, which complements **Ang-1** expression in the basal vessels to balance vessel growth and stabilization. These coordinated, hormone-dependent angiogenic and remodeling processes maintain cyclic vascular integrity and endometrial receptivity for implantation. *Created with* BioRender.com.

The endometrium is a highly dynamic tissue that undergoes cyclical remodeling under hormonal regulation. Histologically, it comprises two distinct layers: the basal layer (*stratum basalis*), a deep, regenerative zone that remains intact after menstruation, and the functional layer (*stratum functionalis*), which proliferates, differentiates, and sheds during each menstrual cycle (9) (Figure 1). The measurable EMT reflects the expansion of the functional layer, encompassing epithelial, stromal, and vascular proliferation (10). As endometrial growth is tightly coupled with vascular development, inadequate angiogenesis or impaired uterine blood flow may constrain stromal expansion and result in a persistently thin endometrium. Moreover, successful implantation and placentation depend on adequate perfusion; hence, vascular insufficiency constitutes a key mechanistic link between poor uterine perfusion, thin endometrium, and reduced reproductive outcomes (11).

**Figure 2:**
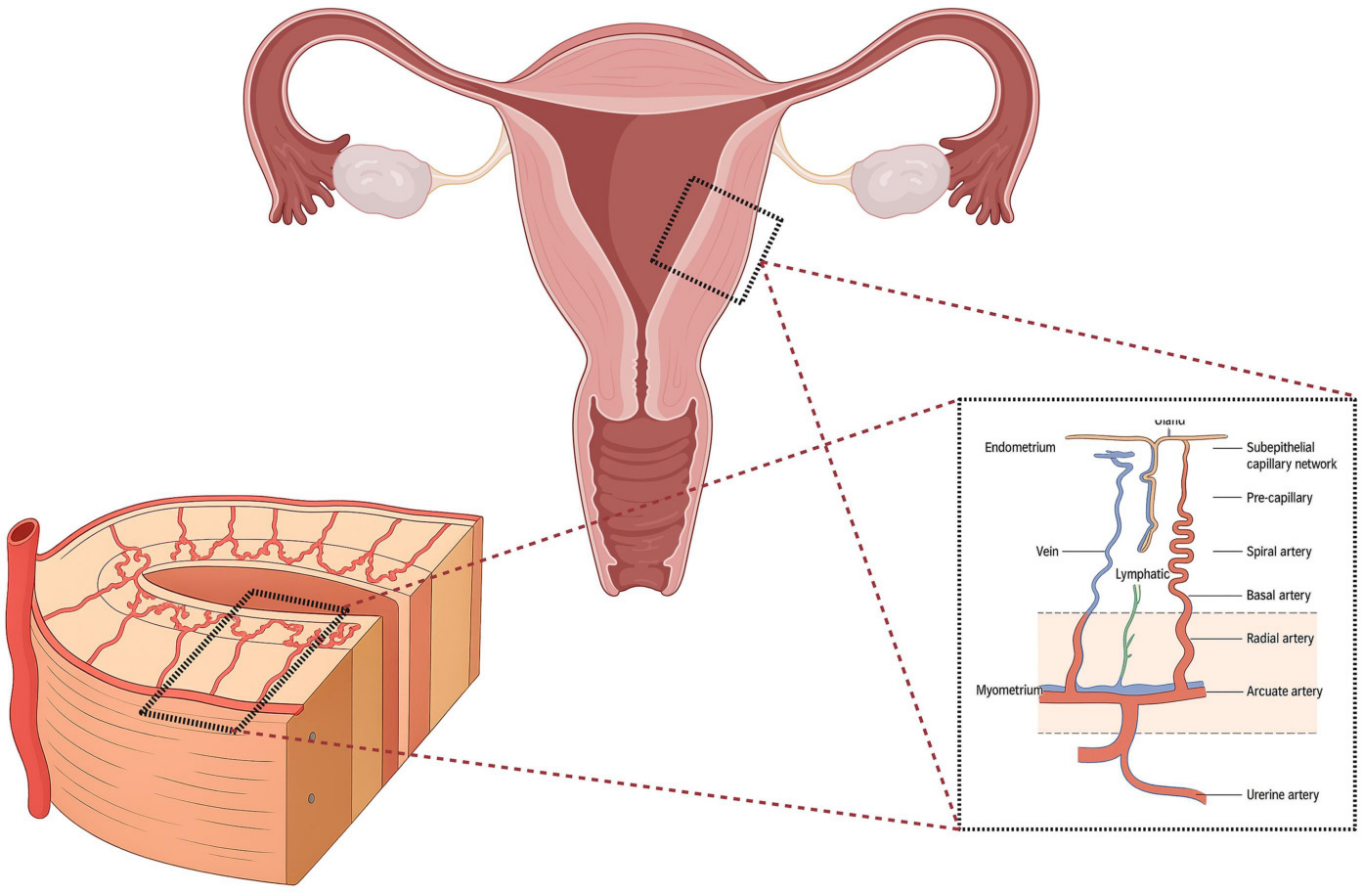
Anatomy of the uterine and endometrial vascular architecture. Illustration of the hierarchical organization of the uterine vasculature and the blood flow pattern within the endometrium. The **uterine artery** branches into **arcuate arteries** coursing circumferentially through the myometrium, which give rise to **radial arteries** penetrating toward the endometrium. Each radial artery divides into **basal** and **spiral arteries** supplying the **basalis** and **functionalis** layers, respectively. The spiral arteries exhibit cyclical elongation, coiling, and regression under hormonal control, and connect to a dense **subepithelial capillary plexus** in the superficial endometrium. The diagram also depicts parallel **venous** and **lymphatic** drainage systems that accompany arterial branches. This intricate vascular network ensures cyclic perfusion, oxygen delivery, and efficient remodeling—processes that are disrupted in conditions associated with **persistently thin endometrium** and impaired receptivity. *Created with* BioRender.com.

As illustrated in Figure 2, the uterine arteries course medially along the uterine wall and give rise to arcuate arteries that encircle the myometrium. From these emerge radial arteries, which penetrate perpendicularly toward the endometrium (12). Near the endometrial–myometrial junction, the radial arteries bifurcate into two systems: basal arteries, supplying the stratum basalis, and spiral arteries, ascending through the stratum functionalis (12, 13). The spiral arteries exhibit marked morphological plasticity across the menstrual cycle. During the proliferative phase, estrogen drives their elongation and coiling, whereas post-ovulation progesterone induces further tortuosity and lumen narrowing, regulating perfusion to the secretory endometrium (13, 14).

Near the luminal surface, the spiral arteries branch into a dense subepithelial capillary plexus, generating the characteristic lacunar vascular pattern observed histologically and in Doppler imaging (15). Venous drainage parallels this organization, with venules coalescing into venous lakes within the functionalis that drain through radial veins into the myometrial arcuate plexus (12).

The endometrial microcirculation is exquisitely hormone responsive. During menstruation, prostaglandins, endothelin-1, and local hypoxia induce vasoconstriction of the spiral arterioles, precipitating ischemic necrosis and shedding of the superficial functionalis (14). Rapid post-menstrual repair follows through robust angiogenesis, mediated by endothelial cell migration and proliferation under the influence of VEGF, FGF, and angiopoietins (13, 16). This recurring cycle of vascular regression and regeneration is unique among adult tissues and fundamental to endometrial physiology (17). Consequently, vascular remodeling in the endometrium deviates from classical angiogenic paradigms as it occurs constantly throughout the reproductive lifespan. Despite extensive tissue turnover and edema, vascular density remains relatively constant throughout the cycle (13), reflecting a tightly controlled equilibrium between vessel formation, regression, and structural adaptation.

Endometrial cells, particularly resident macrophages, secrete a diverse array of angiogenic mediators, including VEGF (18), FGF (19), TGF-α (20), EGF (21), IL-1, IL-6 (22), and IL-8 (23). However, cytokine expression varies markedly among individuals and cycles, and their overlapping functions obscure the exact mechanisms regulating angiogenesis. Clarifying these pathways is key to understanding how vascular dynamics influence endometrial receptivity (Figure 1).

### Pathophysiology and the role of impaired blood supply in persistently thin endometrium

A persistently thin endometrium arises from structural or functional disturbances that reduce its capacity to regenerate and develop blood vessels. While several etiologies exist, many converge on a central pathophysiologic theme: impaired angiogenesis and compromised uterine perfusion. Structural injury to the basal layer, whether through vigorous curettage, intrauterine adhesions (Asherman's syndrome), or repeated uterine surgeries, can destroy the endometrial stem cell niche and its vascular bed, leading to fibrosis and reduced regenerative capacity. Chronic endometritis similarly induces low-grade inflammation, microvascular injury, and stromal fibrosis, further compromising revascularization.

Hormonal factors can also contribute. Clomiphene citrate, a selective estrogen receptor modulator frequently used for ovulation induction, exemplifies iatrogenic endometrial resistance. Its anti-estrogenic action diminishes endometrial perfusion and estrogen-driven angiogenesis, resulting in a thin, poorly vascularized lining (24–27). Although thin endometrium can accompany menopause, primary ovarian failure, or Asherman's syndrome, these may represent distinct pathologies. This review emphasizes idiopathic endometrial resistance, characterized by a thin endometrium (<8 mm) despite adequate hormonal stimulation (28).

Histological and molecular analyses reveal that such endometria are not merely thinner but biologically deficient. In a seminal study, Miwa et al. (10) compared mid-luteal biopsies from women with thin (<8 mm) vs. normal (≥8 mm) EMT. The thin group exhibited markedly reduced vascularity, low VEGF expression, and higher uterine radial artery resistance (RA-RI), indicating a direct link between perfusion and endometrial growth. Similarly, Alfer et al. (28) demonstrated diminished expression of key angiogenic and receptivity markers, including VEGF, β3-integrin, and leukemia inhibitory factor (LIF), in thin endometria (**outlined in** Figure 1). These findings suggest that deficient angiogenic signaling leads to poor tissue proliferation and receptivity, perpetuating a “thin” phenotype.

### Evidence for impaired blood flow as a causal mechanism

A growing body of hemodynamic evidence supports poor endometrial perfusion as a causal factor, rather than a mere consequence, of endometrial thinning. Doppler ultrasonography has shown that women with thin linings consistently exhibit elevated resistance and reduced vascular indices within uterine and subendometrial arteries (10, 29). Using color, power, and pulsed Doppler, perfusion is quantified through indices such as the Resistance Index (RI), Pulsatility Index (PI), and 3D vascular indices, Vascularization Index (VI), Flow Index (FI), and Vascularization–Flow Index (VFI) (11, 30, 31). Elevated RI or PI reflects increased vascular resistance and correlates with both thin endometrium and implantation failure.

Qualitative Doppler findings further substantiate this association. Chien et al. (32) classified endometrial perfusion by the depth of color flow penetration: flow restricted to the subendometrial zone denoted poor vascularity, while flow extending through the entire endometrium correlated with optimal receptivity. In their cohort of over 600 IVF patients, the absence of endometrial blood flow was linked to thinner linings, higher uterine artery resistance, and dramatically lower pregnancy rates (7.5% vs. 48% in women with full endometrial–subendometrial flow). These data underscore the dependence of endometrial growth and implantation potential on adequate perfusion.

Doppler 3D imaging adds quantitative insight, showing that non-conception cycles are characterized by reduced subendometrial VI and FI (29, 33). Collectively, these imaging modalities confirm that hemodynamic insufficiency underlies the thin endometrial phenotype.

### Microvascular and cellular evidence of ischemic endometrium

Histological analyses complement imaging data, revealing reduced microvessel density (MVD) and impaired angiogenic signaling in thin endometrium. Immunostaining for endothelial markers such as CD34 or factor VIII consistently demonstrates lower MVD, reflecting diminished capillary formation (28). At the molecular level, decreased expression of VEGF, β3-integrin, and LIF parallels the reduced vascularity seen by Doppler imaging, while increased levels of hypoxia-inducible factors (HIF-1α, HIF-2α) signal a hypoxic microenvironment (10, 34).

Experimental models further support ischemia as a primary insult. Peng et al. (34) mimicked endometrial ischemia by culturing human endometrial organoids under oxygen–glucose deprivation. These organoids exhibited cytoskeletal collapse, epithelial degeneration, and apoptotic changes consistent with hypoxic injury, accompanied by activation of the RhoA/ROCK stress pathway. Endometrial biopsies from women with thin linings showed similar alterations, including reduced CD34-positive vessels and enhanced HIF expression, linking chronic hypoxia to impaired regeneration.

Beyond limiting angiogenesis, ischemia promotes fibrosis and inflammation, further impeding endometrial repair. Persistent hypoxia stimulates profibrotic mediators and suppresses angiogenic responses, creating a self-perpetuating cycle of poor perfusion and poor growth. Even in hormonally responsive tissue, ischemic endometria fail to upregulate key angiogenic and receptivity factors during the secretory phase (28), indicating a breakdown in the normal endocrine–vascular crosstalk necessary for cyclic renewal.

Collectively, morphological, Doppler, and molecular evidence converge to support the concept that inadequate blood supply is not merely associated with but causally drives the persistence of a thin, unreceptive endometrium.

### Effects of poor perfusion beyond implantation failure

Pregnancies achieved despite the presence of a thin lining are often associated with a poorer outcome. A 2022 study by Ganer-Herman et al. examined placental histology in pregnancies that did occur with a thin endometrium and found a higher incidence of placental malperfusion lesions (suggesting blood flow issues to the placenta) compared to pregnancies from normal EMT (35). Miscarriage risk appears higher in thinner linings in large retrospective studies and meta-analysis (3–5). The association with ectopic pregnancy is well documented: in >8,000 ART pregnancies, EMT < 9 mm carried ∼4-fold higher ectopic risk vs. >12 mm after adjustment, with similar findings in newer analyses using thresholds around 8–9 mm (36). For placenta-mediated disorders, multiple studies and a meta-analysis indicate that thin EMT is associated with hypertensive disorders of pregnancy and fetal growth restriction/small-for-gestational-age, and with lower birthweight overall; these signals persist after multivariable adjustment in large single-center cohorts (37). Emerging obstetric data also suggest that thin endometrium before transfer independently increases the risk of placenta accreta spectrum in ART pregnancies without prior cesarean, with a graded rise in risk below ∼10.9 mm and an especially high odds when EMT < 7 mm (38). While effect magnitudes vary with EMT cut-points, cycle type, and confounding by indication, the preponderance of evidence supports counseling that persistently thin EMT is associated with higher risks of miscarriage, ectopic pregnancy, preeclampsia/HDP, SGA/FGR, and placenta accreta spectrum, warranting closer antenatal surveillance when conception occurs under these conditions (3).

### Therapeutic strategies targeting blood supply

Given the central role of blood supply in endometrial development, a variety of therapeutic approaches have been explored to improve endometrial perfusion and thereby treat thin endometrium. These strategies range from pharmacological vasodilators to regenerative medicine techniques, all aiming to enhance angiogenesis or blood flow in the uterine lining. Below, we summarize key interventions and the evidence of their efficacy:

- **Vasoactive Medications:** Drugs that promote vasodilation or improve blood rheology have been used to increase uterine perfusion. Sildenafil (a nitric oxide-mediated vasodilator) administered vaginally can dilate **existing** uterine arteries; a study reported that vaginal sildenafil significantly decreased uterine artery PI/RI and increased EMT, resulting in higher pregnancy rates compared to controls (39). Similarly, a combination of pentoxifylline (a blood flow enhancer) and vitamin E was tested in thin endometrium patients: it was found to improve Doppler flow indices and slightly increase thickness (40). Low-dose aspirin has also been tried to increase uterine blood flow, though its benefit for thin lining is not clearly proven (41). Overall, these medications are low-risk and may be adjuncts, but often alone they are insufficient to dramatically thicken a stubbornly thin endometrium (7). However, the overall evidence supporting these medications remains limited by small study sizes, inconsistent results, and a lack of strong endorsements from major reproductive medicine guidelines.
- **Hormonal Optimizations:** High-dose estrogen therapy is a first-line approach to encourage endometrial proliferation. In patients with thin endometrium, it is often administered via different routes of administration and in very high doses often reaching supraphysiological concentrations. However, once these efforts have been exhausted other empirical supplemental agents are being employed. Agents like **granulocyte colony-stimulating factor (G-CSF)** have been infused into the uterus in attempts to stimulate growth; some reports showed G-CSF can acutely increase EMT in refractory cases, possibly via promoting angiogenesis and tissue growth, but results are variable. Another hormone-related approach is using **hCG intrauterine infusion** (hCG has angiogenic properties), which in a recent trial improved certain immune parameters and was associated with better pregnancy outcomes in thin endometrium patients (7). These strategies underscore the concept of leveraging endogenous factors that might spur blood vessel formation and endometrial development. Yet, despite promising mechanistic rationale, the clinical data remain heterogeneous and insufficiently robust, and no major reproductive society currently recommends these hormonal adjuncts specifically for thin endometrium management.
- **Platelet-Rich Plasma (PRP) Intrauterine Infusion:** PRP has emerged in recent years as a potential therapy for thin endometrium. PRP is a concentrate of autologous platelets containing abundant growth factors such as PDGF, VEGF, and IGF-1, all of which can promote angiogenesis and tissue regeneration (42). Multiple clinical studies, including small trials and case series, have demonstrated that PRP can promote endometrial thickening in women with refractory thin endometrium (43, 44). PRP's efficacy is further supported by Doppler data: patients often show improved subendometrial blood flow after PRP, evidenced by lower uterine artery RI/PI and higher endometrial vascularity indices: Liu et al., 2021 (45) reported enhanced blood flow with PRP + endometrial mechanical stimulation. Agarwal et al. (46), observed increased subendometrial flow post-PRP. Feng et al. (42) conducted a randomized trial comparing single vs. double PRP infusions in thin endometrium patients. The double-infusion group showed superior outcomes: EMT increased to 8.4 mm vs. 8.0 mm with single PRP, and the clinical pregnancy rate was significantly higher at 48.9% vs. 27.0%. Importantly, Doppler hemodynamic parameters in the double-PRP group indicated better perfusion (e.g., RI and PI were markedly lower, indicating reduced vascular resistance). Importantly, despite early enthusiasm and favorable biological plausibility, PRP has not been widely adopted. Indeed, major guidelines, including the most recent guidance, explicitly recommend against PRP for thin endometrium due to insufficient high-quality evidence and lack of reproducible, well-powered randomized trials (7).
- **Cell-Based Therapies:** Regenerative medicine approaches using stem cells or progenitor cells aim to rebuild the endometrial lining and its vasculature. Autologous bone marrow-derived stem cells or mesenchymal stem cells (MSCs) have been transplanted or infused into the uterus in small pilot studies. Tersoglio et al. (47) treated women with refractory thin endometrium with **endometrial MSCs**, reporting a remarkable increase in mean thickness from ∼5.2 mm to 9.9 mm and a high clinical pregnancy rate of 79%. Umbilical cord MSCs seeded on a collagen scaffold have also been applied to Asherman syndrome patients with thin endometrium, showing regeneration of endometrial tissue and some successful pregnancies (48). These cellular therapies likely work by differentiating into endometrial stromal cells and endothelial cells, secreting growth factors, and generally rejuvenating the basal layer's function including angiogenesis. While unquestionably innovative and promising, cell therapies are still experimental, expensive, complicated and mostly done in specialized centers. They represent the forefront of attempting to permanently restore endometrial vascular networks in women who have lost them due to scarring or other damage. Critically, the evidence base for cell therapies is still restricted to small, early-phase, non-randomized studies, and no professional guidelines support their clinical use outside experimental or trial settings.
- **Hyperbaric Oxygen Therapy**: Emerging data suggest that hyperbaric oxygen therapy (HBOT), may represent a promising adjuvant approach in the management of persistently thin endometrium. In a recent prospective cohort with propensity-matched controls, patients with refractory endometrium (<7 mm) received daily HBOT for at least 10 days during the proliferative phase and achieved a statistically significant increase in EMT (5.76 ± 1.66 mm → 6.57 ± 1.23 mm, *P* = 0.002) alongside higher intrauterine pregnancy rates when blastocyst transfers were performed (53.8% vs. 18.2%, *P* = 0.017) compared to controls (49). Earlier investigations in non-ART settings also noted that HBOT at ∼2.3 ATA improved endometrial sonographic quality, reduced vascular resistance, and increased thickness compared to pre-therapy cycles in infertile women (50). The mechanistic rationale is plausible: HBOT enhances tissue oxygenation, reduces hypoxia-driven angiogenic impairment, improves microvascular perfusion and may restore endometrial receptivity by augmenting neo-angiogenesis (49). Nonetheless, despite these encouraging findings the evidence remains preliminary and of low to moderate quality. Accordingly, HBOT should currently be regarded as investigational in the context of thin endometrium until randomized controlled trials with sufficient power and longitudinal follow-up become available.

Despite encouraging outcomes, the overall efficacy of most therapeutic approaches for thin endometrium remains modest at best. Many strategies share a common rationale: enhancing endometrial blood flow or angiogenic activity, but clinical results have been inconsistent. A recent network meta-analysis of 18 randomized controlled trials compared six commonly investigated interventions: oral aspirin, Ding Kun Dan, intrauterine platelet-rich plasma (PRP), intrauterine granulocyte–macrophage colony-stimulating factor (G-CSF), intramuscular recombinant human growth hormone (rhGH), and neuromuscular electrical stimulation (NMES) (51). Among these, PRP ranked highest for improving EMT and clinical pregnancy rates. Nevertheless, given the limited absolute benefit observed even with PRP, the effectiveness of the other modalities appears even less compelling. In fact, the most recent practice guidelines on the management of IVF cycle associated with thin endometrium the authors either do not support or recommend against the use of all the previously mentioned interventions due to lack of efficiency (7).

Historically, most therapeutic strategies for persistently thin endometrium were not conceived with the central premise that impaired endometrial perfusion is the core pathophysiological defect. Nevertheless, the few interventions that have demonstrated some degree of clinical benefit, those currently incorporated into the therapeutic armamentarium, are precisely the ones that incidentally enhance endometrial blood flow. Despite this, these modalities are often cumbersome, invasive, variably effective, and sometimes associated with adverse effects, ultimately providing only modest clinical improvement. As the pivotal role of deficient endometrial vascularization becomes increasingly clear, there is a pressing need to develop targeted, mechanism-based therapies rooted in contemporary understanding of angiogenic regulation. Such approaches have the potential to yield substantially greater efficacy while reducing treatment burden and improving patient safety.

### Limitations of the current database

While considerable evidence supports poor blood supply as a key driver of thin endometrium, there are important limitations and gaps in the current knowledge:

#### Study limitations

Many studies on this topic are small, non-randomized, or heterogeneous in design. Definitions of “thin endometrium” vary between studies (some use <6 mm, others <7 or <8 mm), making it challenging to compare results across the literature. Additionally, most intervention studies lack long-term follow-up; increased thickness is often reported, but it is sometimes unclear if this translates to higher live birth rates, as not all trials are powered for clinical pregnancy or live birth outcomes. There is also a publication bias tendency, positive results (showing improved thickness/pregnancy) are more likely to be reported, whereas failures of interventions may go unreported. As such, the field would benefit from larger, controlled trials to confirm which perfusion-targeting therapies truly impact live birth rates in a cost-effective manner.

#### Mechanistic uncertainties

Although hypoxia and angiogenic impairment have been demonstrated in thin endometrium (28, 34), the precise molecular pathways linking poor perfusion to endometrial unresponsiveness are not fully delineated. For instance, are there specific gene expression changes in endothelial cells or stromal cells due to chronic low blood flow that could be targeted by drugs? Single-cell analyses and transcriptomic studies of thin vs. normal endometrium could shed light on which cell types are most affected (e.g., endothelial cells failing to proliferate, stromal cells becoming fibrotic).

#### Patient heterogeneity

Women with thin endometrium form a heterogeneous group. Some have underlying Asherman syndrome, some have normal uterine anatomy but thin lining during IVF (perhaps related to unmodifiable factors like age or unrecognized basal layer deficiencies), and others have iatrogenic thin endometrium from prolonged hormonal treatments. The optimal approach to improve blood supply might differ between these scenarios. For example, Asherman syndrome might respond best to stem cell regenerative therapy, whereas an idiopathic thin endometrium in an IVF cycle might respond to PRP or vasoactive drugs. Future research should stratify patients to tailor therapies appropriately.

## Conclusion

Cumulative evidence from histological, hemodynamic, and molecular studies supports impaired uterine perfusion as a central pathophysiologic mechanism underlying persistently thin endometrium. Insufficient angiogenesis, increased vascular resistance, and hypoxia impair stromal growth, implantation, and placental development, predisposing to miscarriage and placental dysfunction and the related pregnancy complications.

Existing pharmacologic, biologic, and physical treatments remain empirical, offering only short-term marginal gains without consistent improvement in live-birth outcomes.

Future progress depends on mechanistically designed, angiogenesis-targeted interventions validated through well-controlled trials and standardized perfusion assessment, to achieve true restoration of endometrial vascular integrity and function.
